# Mechanisms of Xiaochaihu Decoction on Treating Hepatic Fibrosis Explored by Network Pharmacology

**DOI:** 10.1155/2022/8925637

**Published:** 2022-10-04

**Authors:** Rui Qiang, Ya Zhang, Yanhong Wang

**Affiliations:** ^1^Guang'anmen Hospital of China Academy of Chinese Medical Sciences, Beijing 100053, China; ^2^Shaanxi University of Chinese Medicine, Xianyang 712046, China; ^3^Department of Orthopedics, Tongji Hospital, School of Medicine, Tongji University, Shanghai 200065, China; ^4^Key Laboratory of Spine and Spinal Cord Injury Repair and Regeneration, Ministry of Education, Shanghai 200065, China

## Abstract

**Purpose:**

To explore the material basis and pharmacological mechanism of Xiaochaihu Decoction (XCHD), the classic Traditional Chinese Medicine (TCM) formula in inhibiting hepatic fibrosis (HF).

**Methods:**

The main components in XCHD were screened from the TCMSP database, ETCM database, and literature, and their potential targets were detected and predicted using the Swiss Target Prediction platform. The HF-related targets were retrieved and screened through GeneCard database and OMIM database, combined with GEO gene chips. The XCHD targets and HF targets were mapped to search common targets. The protein-protein interaction (PPI) network was acquired via the STRING11.0 database and analyzed visually using Cytoscape 3.8.0 software. The potential mechanisms of the common targets identified through GO and KEGG pathway enrichment analysis were analyzed by using Metascape database. The results were visualized through OmicShare Tools. The “XCHD compound-HF target” network was visually constructed by Cytoscape 3.8.0 software. AutoDockVina1.1.2 and PyMoL software were used to verify the molecular docking of XCHD main active compounds and HF key targets.

**Results:**

A total of 164 potential active compounds from XCHD were screened to act on 95 HF-related targets. Bioinformatics analysis revealed that quercetin, *β*-sitosterol, and kaempferol may be candidate agents, which acted on multiple targets like PTGS2, HSP90AA1, and PTGS1 and regulate multiple key biological pathways like IL-17 signaling pathway, TNF signaling pathway and PI3K-Akt signaling pathway to relieve HF. Moreover, molecular docking suggested that quercetin and PTGS2 could statically bind and interact with each other through amino acid residues val-349, LEU-352, PHE-381, etc.

**Conclusion:**

This work provides a systems perspective to study the relationship between Chinese medicines and diseases. The therapeutic efficacy of XCHD on HF was the sum of multitarget and multi-approach effects from the bioactive ingredients. This study could be one of the cornerstones for further research.

## 1. Background or Introduction

Hepatic fibrosis (HF) is a reversible liver damage before various chronic liver diseases develop into irreversible liver injuries such as cirrhosis or even liver cancer. It is a key stage to affect the prognosis of chronic liver diseases [1]. The pathomechanism of HF is varied, including hepatitis virus infection, alcoholism, drug abuse, parasites, metabolic disorders, cholestasis, and immunologic derangement. If the liver injury is temporary, fibrosis can be gradually absorbed, and normal structure of liver can be restored. But in severe cases, the extracellular matrix (ECM) will gradually accumulate and become uncontrollable, then extend into the intercellular space of liver, and gradually replace liver tissues. The normal physiological function of liver has affected, and the risks of liver-related complications have increased during this process, such as cirrhosis and hepatocellular carcinoma [2, 3]. It has to be acknowledged that HF, as a refractory disease, remains a formidable health problem all over the world. The current treatment mainly involves two aspects: One is the treatment of etiology. Nonetheless, etiological treatment also has limitations, such as failure to completely suppress inflammation [4], and there are certain etiology-unknown chronic liver diseases. The second treatment is aimed at HF. Unfortunately, once the pathomechanism of HF is activated, it often presents a complex network involving multigenes, multicells, and multifactors. Because most drugs have a single mechanism of action with certain unwanted side effects, modern medicine does not have a particularly suitable and specific treatment for HF. Traditional Chinese Medicine (TCM) has its oneself unique advantages and characteristics of multicompound, multitarget, multipathway, personalized treatment with fewer toxic side effects. It has been the most frequently used additional complementary and alternative medicines for HF patients. TCM has been used in the whole process of prevention, treatment and reverse of HF [5].

TCM has almost 5000 years of history, which creates many treatments for disease. Xiaochaihu Decoction (XCHD) is a classic Chinese medicine formula pioneering by the medical sage Zhong-Jing Zhang [6], and it was originated from the book of Treatise on Febrile Diseases (Shang Han Lun) in the Han Dynasty (200–201 AD) China. It has a history of more than 1800 years with a curative effect in treating chronic liver diseases. XCHD is a concoction made of Chinese herbal medicines, including 24 g Radix Bupleuri (Chinese thorowax root), 9 g Radix Scutellariae (huangqin or baical skullcap root), 9 g Rhizoma Pinelliae (banxia or pinellia tuber), 9 g Radix Ginseng (renshen or ginseng), 9 g Radix Glycyrrhizae (gancao or licorice root), 9 g Rhizoma Zingiberis Recens (shengjiang or fresh ginger), and 40 g Fructus Jujubae (dazhao or Chinese date). Massive experimental evidence has demonstrated that XCHD protects liver from injuries [7–11], prevents, and treats hepatic and pancreatic fibrosis [12–14]. Despite XCHD's long history of clinical use and abundant experimental support, its active ingredients, putative target genes, and underlying mechanism have not been fully clarified.

**Figure 1:**
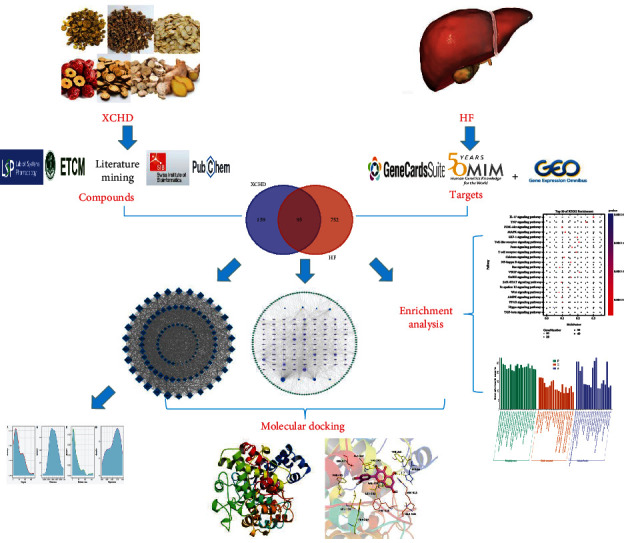
The research flow chart.

Network pharmacology is an emerging and promising interdisciplinary subject. It is the combination of classical pharmacology, molecular biology, bioinformatics, and computer technology. It can avoid problems of high cost and long cycle of experiments and drug research. Its concept of holism, systems, and interaction are consistent with the characteristics of TCM treatment. It can systematically explain the complex mechanism of TCM prescriptions on treating refractory diseases [15] and provide the crucial support for clinical utilization. It also has been considered the next paradigm in drug discovery [16]. Additionally, molecular docking is a theoretical method to study the interaction and recognition between protein receptors and small molecule ligands [17–19]. It has become an important technology in the field of computer-aided drug research [20]. Collectively, network pharmacology and molecular docking have successfully predicted the TCM mechanism in treatment of many serious and complex diseases. In this study, the pharmacological mechanism of XCHD in the treatment of HF, which we explored, was approached by microarray analysis combined with network pharmacology and molecular docking. The research flowchart is shown in Figure 1.

## 2. Methods

### 2.1. Research Tools

Data were analyzed in R version 4.2.0. Cytoscape was used to visualize the PPI network (version 3.8.0) 3.8.0 [21]. The rest of research tools and web sites were as follows: Gene Expression Omnibus (https://www.ncbi.nlm.nih.gov/geo/), Traditional Chinese Medicine Systems Pharmacology Database and Analysis Platform (TCMSP, http://lsp.nwu.edu.cn/tcmsp.php) [22], The Encyclopedia of Traditional Chinese Medicine (ETCM, http://www.tcmip.cn/ETCM/index.php/Home/), PubChem (https://pubchem.ncbi.nlm.nih.gov/), Online Mendelian Inheritance in Man (OMIM, https://omim.org/), Swiss Target Prediction (http://www.swisstargetprediction.ch/) [23], Uniprot (http://www.uniprot.org/) [24], GeneCards (https://www.genecards.org/, Version:5.0) [25], Venny 2.1 (https://bioinfogp.cnb.csic.es/tools/venny), STRING11.0 (https://string-db.org) [26], Metascpe (http://metascape.org/gp/index.html) [27], OmicShare Tools (http://www.omicshare.com/), Protein Data Bank (PDB) (https://www.rcsb.org/), AutoDockVina 1.1.2, and PyMoL [28].

### 2.2. Screening for Active Ingredients in XCHD

The chemical constituents of XCHD were screened by TCMSP database, ETCM database, and literature mining. OB value (systemic bioavailability after oral absorption and distribution) ≥30% and DL value (structural similarity between compounds and clinically used drugs in the DrugBank database) ≥0.18 were selected as screening criteria. With the help of PubChem, we plotted the two-dimensional structure of main components, which was inputted into Swiss Target Prediction to predict the targets of the main components based on the similarity of two-dimensional structure. All target proteins of each drug are inputted into the Uniprot database to search for corresponding genes. The search criterion was that " homosapens (Human)" was set as the background organism. These data were used to construct the compound-target protein-gene interaction network.

### 2.3. Identification of Potential Targets for HF

The gene expression profiles of HF (GSE197112) were downloaded from the Gene Expression Omnibus (GEO) database, and “GEOquery,” “Limma,” and “AFFy” packages of R4.2.0 language were used to convert the downloaded probes into corresponding gene names and obtain upregulated and downregulated differentially expressed genes (DEGs), which were saved in Excel. DEGs with the cut-off criteria of *P* value < 0.005 and |log2 (fold change)| > 1 were visualized by volcano map and heat map. In addition, GeneCards database and OMIM database provided target information related to HF-related disease. Finally, through searching keywords based on MeSH/PubMed: Hepatic Cirrhosis?Cirrhosis, Hepatic?Cirrhosis, Liver?Fibrosis, Liver?Liver Fibrosis etc, the targets of HF-related disease were collected. We then combined those results with GEO analysis results to get HF-related genes.

### 2.4. Construction of the PPI Network and Topological Analysis

XCHD and HF targets were matched one by one, entered into Venny 2.1 software, and these two overlapping targets were uploaded to STRING11.0 database to construct the protein-protein interaction (PPI) network. The species was named as Homo sapiens. Set the minimum interaction threshold to “Maximum confidence” (>0.4) and the rest to default values. In Cytoscape 3.8.0, overlapping targets were visually constructed for active components-target network of XCHD and HF. The CytoNCA plugin in Cytoscape3.8.0 software was utilized to calculate topological parameters of nodes, including degree, obligatory, betweenness, and eigenvector, all of which can provide an in depth analysis of the attributes of nodes in the interactive network.

### 2.5. GO and KEGG Enrichment Analysis

The intersection of XCHD and HF targets was recorded on Metascape platform and set up *P* < 0.01, while the main biological processes and metabolic pathways were analyzed and enriched. The obtained information was processed and imported into OmicShare Tools for analyzing and visualizing the results.

### 2.6. Molecular Docking

Based on the above results, the active components of the top three in degree were used as ligands, and the targets of the top three in degree were used as receptors for molecular docking. The target protein structure was received from the PDB database, and the three-dimensional structure of the active ingredients downloaded from PubChem CID were imported into AutoDockVina1.1.2 and PyMoL software, and search for the optimal conformation.

## 3. Results

### 3.1. Active Components in XCHD

We relied on TCMSP, ETCM database, and literatures to search for the chemical constituents of XCHD. A total of 17 species of Bupleurum, 13 species of Pinellia, 36 species of Baicalensis, 22 species of ginseng, 92 species of licorice, 29 species of jujube, and 5 species of ginger were obtained. All chemical compounds were screened, and the duplicated compounds without corresponding targets were deleted. Then, we obtained 164 potential active compounds from XCHD for further analysis. According to the OB value, the basic information of the top 20 active components in XCHD is listed in Table 1.

### 3.2. Potential Target Prediction of HF

**Figure 2:**
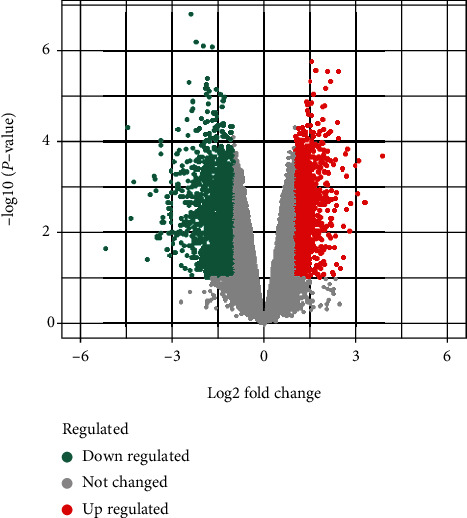
Volcanic map of differential genes between normal subjects and patients with HF. Each dot represents a gene. Gray dots refer to genes with no difference in expression between normal people and patients with HF, green dots are downregulated genes, and red dots are upregulated genes.

**Figure 3:**
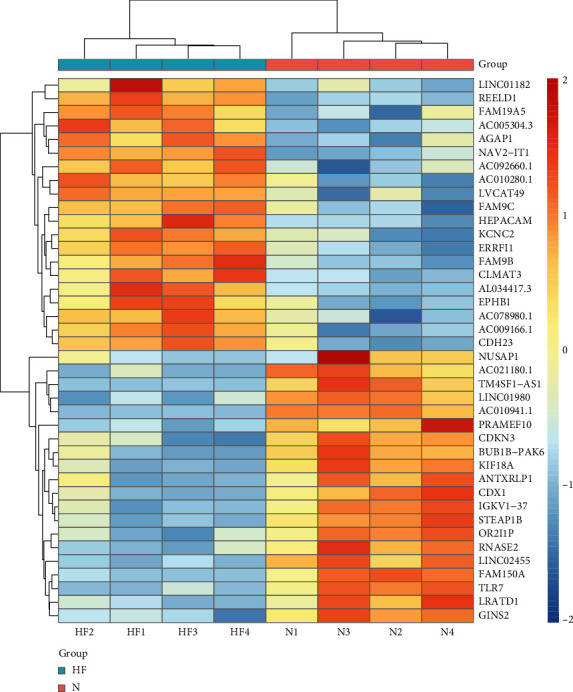
Heat map of differential genes between normal subjects and patients with HF. In the label, pink N represents the normal group, and blue HF represents the HF patient group. The more upregulated the gene, the more red the color. The lower the downregulation, the more blue the color, with yellow representing the genes expressed in the middle.

The gene expression profiles of HF (GSE197112) were consisted of 8 samples. A total of 41489 genes were obtained, including 3750 DEGs, 1848 upregulated genes, and 1902 downregulated genes. In the volcano map (Figure 2), DEGs in the disease samples displayed a normal distribution, with a larger number of significantly upregulated genes than the downregulated ones. The top 20 genes are presented in Figure 3. In addition, we integrated GeneCards database and OMIM database disease targets and combined them with the GEO results to eliminate duplicates, resulting in the identification of 847 HF targets.

### 3.3. Construct and Analysis the PPI Network

**Figure 4:**
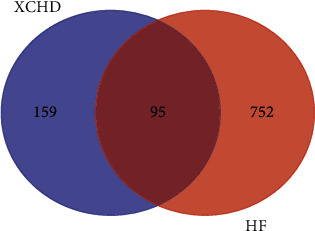
Venn diagrams of potential XCHD targets and HF-related targets. 95 overlapped genes of XCHD targets and HF targets were identified.

**Figure 5:**
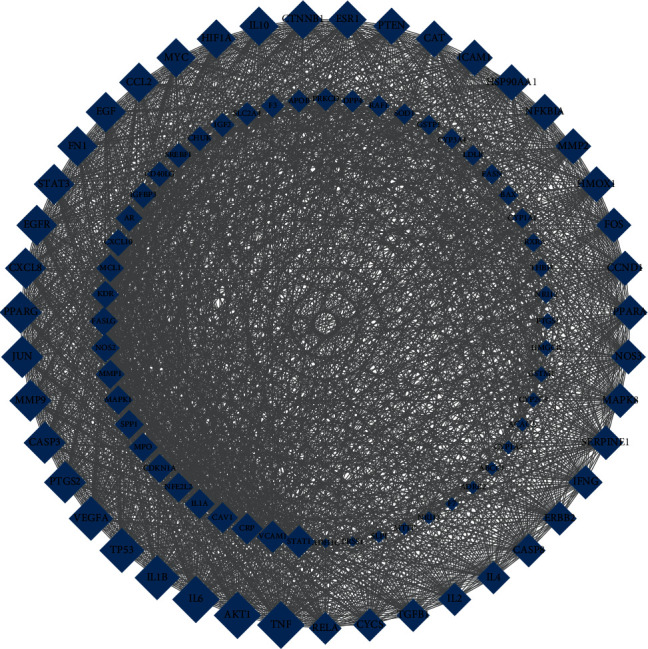
The key targets of XCHD and PPI network of HF. Arrange 95 nodes in order according to degree value. The top six targets were TNF, AKT1, IL6, TP53, IL1B, and VEGFA. There were 95 nodes, 2100 edges, and the average node degree was 44.1.

**Figure 6:**
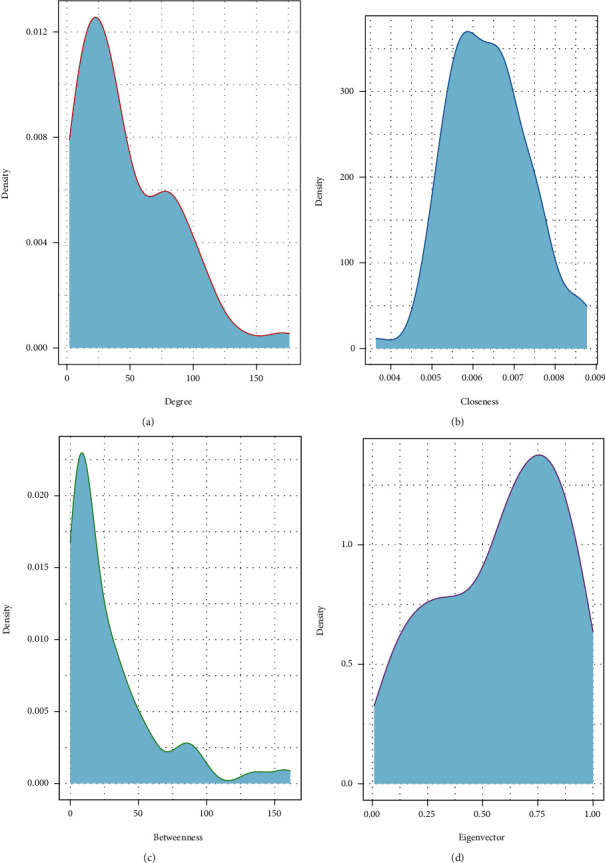
Analysis of PPI network topology. (a) Degree distribution of network, (b) closeness centrality distribution of network, (c) betweenness centrality distribution of network, (d) eigenvector centrality distribution of network.

According to the selected XCHD active components and HF targets, Venny 2.1 online software was used to draw Venn diagram, and 95 overlapped genes of XCHD targets and HF targets are identified in Figure 4. The overlapped genes were submitted to STRING11.0 database to construct PPI network, and the results were visualized by Cytoscape 3.8.0 software in Figure 5. The inclusion criteria of “degree” ≥2 times of the median and “closeness centrality” ≥ median (Table 2). Finally, an analysis of topological parameters including degree, closeness, betweenness and eigenvector is carried out in Figure 6.

**Figure 7:**
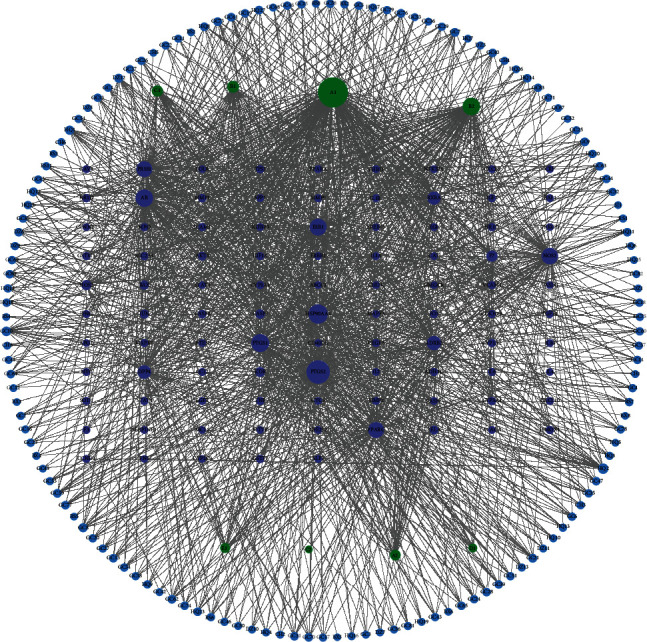
XCHD compound-HF target network diagram. The blue nodes represent the main active components of XCHD, and the purple nodes represent the corresponding targets, the green nodes represent compounds common to different drugs.

We constructed a map of the main active components of XCHD and their corresponding targets (Figure 7). Among the three main active components, the highest degree was quercetin (A1, degree value 217), the second was kaempferol (B2, degree value 95), and the third was *β*-sitosterol (B1, degree value 42). These may be essential active components with the therapeutic potential in XCHD for HF. The three corresponding maximum targets were PTGS2 (degree value 154), HSP90AA1 (degree value 114), and PTGS1 (degree value 101). In addition, according to the topological parameters calculated by Cytoscape3.8.0 software, the average degree was 15.58 of XCHD active compound-target network in treating HF. The top 10 topological parameters of active components and targets in XCHD are sorted in Table 3 and Table 4.

### 3.4. GO Analysis and KEGG Pathway Analysis

**Figure 8:**
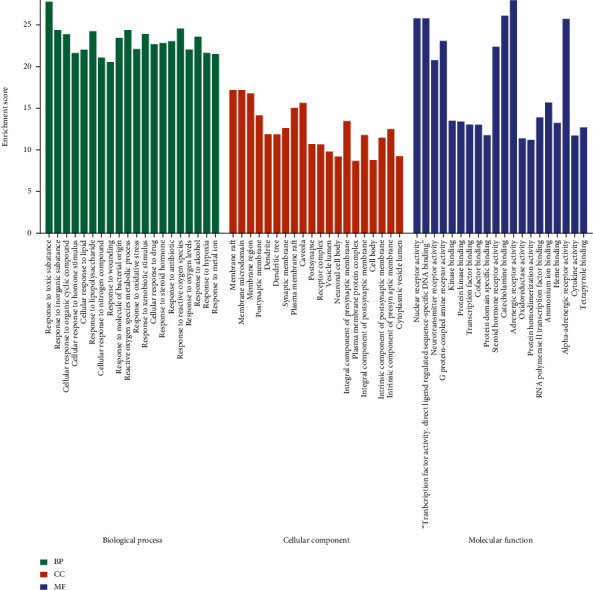
GO enrichment analysis. Sorted by the degree of significance, the BP is significantly enriched in biological response to lipopolysaccharide, MAPK cascade regulation, apoptosis signaling pathway, reactive oxygen metabolism, cell adhesion regulation and positive regulation of cell death, etc. MF is mainly enriched in protein binding, protein kinase binding, enzyme binding region, and transcription factor binding, mainly involving protein and transcription factor activity, etc. CC is mainly enriched in membrane raft, postsynaptic membrane, dendrite and receptor complex, etc.

**Figure 9:**
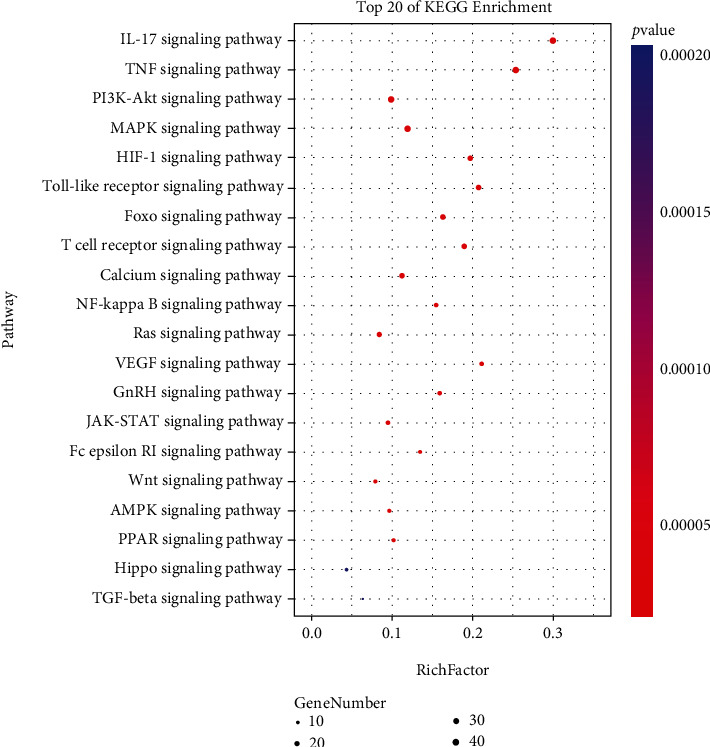
KEGG pathway analysis. The area of the bubble represents the number of enriched genes in the pathway, and the color of the bubble represents the size of the *P* value. The color from red to blue reflects the *P* value from large to small, and the bubble from small to large reflects the number of enriched genes from small to large. The greater the Rich factor value, the greater the degree of enrichment.

Metascape website was used to conduct GO analysis and KEGG pathway analysis on the targets of XCHD in treating HF. The GO analysis consisted of biological processes (BP), cellular compound (CC), and molecular function (MF). The results showed that the 95 genes were enriched in 2423 GO entries (*P* < 0.05), including 2026 BP, 134 CC, and 263 MF. KEGG pathway analysis obtained 46 pathways and the main components of XCHD in the treatment of HF were enriched in signaling pathways such as IL-17 signaling pathway, TNF signaling pathway, and PI3K-Akt signaling pathway. XCHD compounds may act on these pathways to against HF. The top 20 significant enrichment terms of BP, CC, and MF and the top 20 significant KEGG signaling pathways were imported into OmicShare Tools to visually analysis and showed in Figures 8 and 9.

### 3.5. Molecular Docking

**Figure 10:**
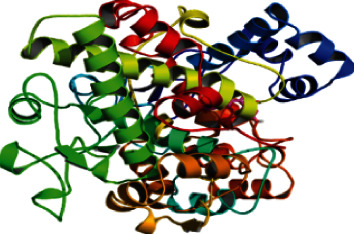
Quercetin docking with PTGS2 (overall picture). Quercetin binds to the active pocket of PTGS2 in a compact conformation and interacts with surrounding amino acids.

**Figure 11:**
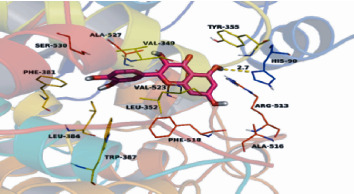
Quercetin docking with PTGS2 (detailed picture). Quercetin is located in the hydrophobicity formed by amino acid residues Val-349, Leu-352, Phe-381, Leu-384, Trp-387, Ala-516, Phe-518, Leu-523, and Ala-527 Cavity pockets and forms strong hydrophobic interactions. Detailed analysis shows that the chromone backbone of quercetin can form a cation-*π* interaction with the side chain of amino acid residue Arg-513. Importantly, the hydroxyl group of quercetin can interact with the amino acid residue His-90 to form a 2.7 Å hydrogen bond, which is the most significant binding force between quercetin and PTGS2. All these interactions make the quercetin form a stable complex with PTGS2.

By using AutoDockVina1.1.2 and PyMoL software, the three target proteins (PTGS2, HSP90AA1, and PTGS1) with the larger degree and the three compounds with more predicted targets in active components targets network of XCHD and HF were, respectively, molecularly docked. The molecular docking results showed that the lowest binding energy of the molecule and the target protein were less than 0, indicating that the ligand and the receptor could spontaneously bind. The docking binding energy of quercetin of PTGS2, HSP90AA1, and PTGS1 was all less than-5 kcal·mol-1. The binding energy was, respectively, − 8.5, − 7.6, and − 7.0 kcal·mol^−1^(Table 5). The lower the binding energy, the higher the affinity of the receptor and the ligand, the more stable the conformation. In order to clarify the mode of the quercetin and human PTGS2 at the molecular level, we docked quercetin to the active pocket of PTGS2 with an affinity of − 8.5 kcal·mol^−1^. These results are showed in Figures 10 and 11.

## 4. Discussion

XCHD is a classic herbal formula with a long history of clinical application. Although the anti-HF role of XCHD has been studied through experiments, systematic research on the exact mechanism of action is lacking, which greatly restricted the modernization and the internationalization of XCHD. As for this concern, we made a combination of bioinformatics analysis, network pharmacology, and molecular docking might contribute to find the therapeutic mechanism of XCHD on HF. Based on data mining, data analysis, and virtual computing, network pharmacology presents the relationships between active ingredients, key targets, related pathways, and diseases in computational modeling analysis. Compared to traditional pharmacology research methods, network pharmacology has shifted the paradigm from a “one-target, one-drug” mode to a “network-target, multiple-component-therapeutics” mode. This approach makes it very powerful and comprehensive for the analysis of drug combinations. Molecular docking can accurately predict the conformation of small molecule ligands within the appropriate target binding site and to assess the binding affinity. In recent years, molecular docking is quickly becoming one of the key component of the drug discovery pipeline.

In this work, a total of 164 potential active compounds from XCHD were screened to act on 95 HF-related targets. Quercetin, kaempferol, and *β*-sitosterol were the top three promising active ingredients, which could act on multiple targets like PTGS2, HSP90AA1, and PTGS1 and regulate multiple biological pathways like IL-17 signaling pathway, TNF signaling pathway, and PI3K-Akt signaling pathway to relieve HF.

Quercetin is an effective liver protectant [29]. It is the major representative of the flavonoid subgroup of flavones with antiviral, anti-inflammatory, antioxidant, antifibrotic, and anticancer pharmacological activities [30]. Activation of hepatic stellate cell (HSC) is a core event of HF and a major factor of collagen deposition. Studies have shown that quercetin can inhibit HSCs activation in vivo and in vitro [31]. Quercetin can decrease the serum biomarker enzymes alanine transaminase (ALT) and aspartate transaminase (AST), as well as alkaline phosphatase (ALP) and total protein in rats with carbon tetrachloride– (CCL_4_–) induced HF. Quercetin can also markedly reverse the histopathological changes in rats with CCL_4_-induced HF [32]. Moreover, it can inhibit the activation of nuclear transcription factor-*κ*B (NF-*κ*B). The inhibition of NF-*κ*B phosphorylation can protect against hepatic injury, inflammation, and fibrosis [33]. Quercetin can also downregulate BCL2-associated X protein (Bax) and upregulate B-cell lymphoma-2 (Bcl-2) in a dose-dependent manner to prevent apoptosis of liver cells. In this way, quercetin can protect liver and attenuate HF [34]. In addition, researches have shown that quercetin is an effective antioxidant and activator of superoxide dismutase and catalase, which can prevent and improve HF by eliminating oxygen free radicals to maintain oxidative balance [35, 36]. Quercetin can alleviate HF by inhibiting macrophage infiltration and targeting Notch1 pathway, which regulates polarization of M1 macrophages [37]. Quercetin can repair the hepatic lipid metabolism disorder, improve the liver steatosis, and delay the progression of non-alcoholic steatohepatitis to HF [38–40]. Quercetin is also an effective therapeutic strategy for concanavalin A-induced autoimmune hepatitis and HF [41]. Furthermore, investigations have confirmed that quercetin has certain preventative and therapeutic effects on a multitude of fibrotic diseases [42].

*β*-sitosterol, which widely exists in natural plants, is classed as phytosterol with multiple biological activities and known as the “key of life” [43]. *β*-sitosterol realizes a downregulation of the mRNA, protein expression of collagen-1, and *α*-smooth muscle actin (*α*-SMA) in activated HSCs, prevents collagen accumulation, alleviates dimethylnitrosamine- (DMN-) induced liver injury in mice, and plays an anti-fibrosis role [44]. It has been reported that *β*-sitosterol has a dose-dependent preventative effect of liver on CCL_4_-induced chronic liver disease, which can inhibit oxidative stress, reduce the expression of HSCs activation markers, alleviate pathological damage of liver, and inhibit fibrosis [45]. Transforming growth factor-*β* (TGF-*β*) is a key mediator of HSCs activation and extracellular matrix deposition, which could result in HF. It is a key inducer of fibrosis in many organs. The severity of HF is positively correlated with TGF-*β* level, and the TGF-*β* pathway is considered as a potential anti-HF target [46].

Kaempferol has been proved to significantly reduce TGF-*β* expression and ECM deposition and relieve HF. Furthermore, intraperitoneal injection of kaempferol significantly reduces necrosis of liver tissues and collagen deposition. The levels of ALT, AST, LN, and HA in kaempferol treatment group were significantly lower than control group. Kaempferol can inhibit collagen synthesis and HSCs activation in vivo and in vitro [47]. Investigations have shown that kaempferol can protect normal liver cells from cytotoxicity induced by hydrogen peroxide [48]. Kaempferol also protects the liver from alcohol-induced damage by reducing the activity and expression of cytochrome 2E1.15 [49].

KEGG pathway analysis shows that the XCHD mechanism of inhibiting HF is related to the IL-17 signaling pathway, TNF signaling pathway, PI3K-Akt signaling pathway, and others. The IL-17 signaling pathway promotes the production of interleukin (IL)-6, IL-1*β*, and tumor necrosis factor-*α* (TNF-*α*) and increases the expression of the main fibrotic cytokine TGF-*β* [50]. In addition, the IL-17 signaling pathway can also stimulate the activation and proliferation of macrophages and HSCs and promote remodeling of liver tissues and the formation of fibrosis [51]. Tumor necrosis factor (TNF) is a powerful inflammatory factor which can proliferate and migrate by itself. It can be combined with IL-6, IL-1*β*, and other cytokines to stimulate the activation of HSCs, induce the appearance of all kinds of fibrotic factors including itself, and form a signal network to jointly affect the initiation and occurrence of HF [52, 53]. The PI3K-Akt signaling pathway plays a vital role in regulating cells proliferation and maintaining the biological characteristics of malignant cells. Akt activated by P13K phosphorylates a series of substrates, thereby affects cell growth, cell cycle, cell differentiation, survival, metabolism, angiogenesis, migration, apoptosis, and other processes and plays a critical role in fibrotic diseases [54]. Studies have confirmed that blocking the PI3K-Akt signaling pathway can inhibit the activation and proliferation of HSCs, facilitate HSCs apoptosis, and reduce the number of HSCs and the deposition of collagen. In brief, inhibition of PI3K-Akt signaling pathway is considered an effective way to treat HF [54–56].

Molecular docking results show that the active compounds of XCHD have good affinities with PTGS2, HSP90AA1, and PTGS1. Prostaglandin-endoperoxide synthase (PTGS/COX) is a key rate-limiting enzyme in the synthesis of prostaglandins from arachidonic acid. Two isoenzymes of this enzyme, PTGS1 (COX-1) and PTGS2 (COX-2), have been identified. PTGS1 (COX-1) mainly acts on endoplasmic reticulum and expresses in many tissues. It is a constituent enzyme involved in physiological processes. PTGS2 (COX-2) mainly acts on cell membranes, and its expression induced by a series of stimuli is a major compound of inflammation and HF [57, 58].Under normal physiological conditions, PTGS2 (COX-2) is almost unexpressed in the liver, but excessive expression can be induced under the stimulation of various physical and chemical factors [59]. The excessive expression of PTGS2 can regulate the proliferation and apoptosis of HSCs, increase the fibrogenic factors, and reduce the degradation of ECM. Its expression affects the severity of various liver diseases [60, 61]. As a consequence, XCHD might play an anti-HF role by regulating PTGS1, PTGS2, and other corresponding targets, thus attenuating the inflammatory response of liver. Heat shock protein 90 (HSP90) is a highly conserved molecular chaperone. HSP90 inhibitor induces HSCs apoptosis via a sphingomyelinase- and NF-*κ*B-dependent mechanism [62]. Inhibition of HSP90 also reduces the expression of IL-1*β* and IL-18 [63] and mitigates liver inflammation. HSP90AA1, a subtype of HSP90, has been reported as a very attractive therapeutic target for HF [64].

## 5. Conclusions

The occurrence and development of HF is a complex biological process involving multiple targets and multiple pathways. Without effective treatment, HF might transform into cirrhosis or even liver cancer, greatly affecting the patient's quality of life and life period. Hence, it is of great significance to intervene in the pathological progression of HF. This study preliminarily proved that the therapeutic efficacy of XCHD on HF was the sum of multitarget and multi-approach effects from the bioactive ingredients. The results also provide more references for XCHD' clinical application and development, whereas the results of this work also have limitations. The present research data from different online databases was based on the reviewed and predicted data. Thus, those unproven, undocumented, and unupdated compounds or targets may not be included in our analysis. In a next step, we will carry out in vivo and in vitro experiments focusing on the stability test, pharmacodynamic evaluation, omics techniques, and pathway validation to conduct in-depth observations on the mechanism of the XCHD in treating HF.

## Figures and Tables

**Table 1:** Basic information of active compounds in XCHD.

MOLID	Compound	OB	DL	Source	Symbol
MOL002934	Neobaicalein	104.34	0.44	Scutellariae radix	HQ15
MOL002311	Glycyrol	90.78	0.67	Glycyrrhizae radix	GC3
MOL012992	Mauritine D	89.13	0.45	Zizyphi Fructus	DZ5
MOL004990	7,2′,4′-trihydroxy-5-methoxy-3-arylcoumarin	83.71	0.27	Glycyrrhizae	GC69
MOL004904	Licopyranocoumarin	80.36	0.65	Glycyrrhizae	GC43
MOL004891	Shinpterocarpin	80.3	0.73	Glycyrrhizae	GC40
MOL005017	Phaseol	78.77	0.58	Glycyrrhizae	GC81
MOL004841	Licochalcone B	76.76	0.19	Glycyrrhizae	GC26
MOL002932	Panicolin	76.26	0.29	Scutellariae	HQ13
MOL004810	Glyasperin F	75.84	0.54	Glycyrrhizae	GC14
MOL001484	Inermine	75.18	0.54	Glycyrrhizae	GC1
MOL000500	Vestitol	74.66	0.21	Glycyrrhizae	GC73
MOL012246	5,7,4′-trihydroxy-8-methoxyflavanone	74.24	0.26	Scutellariae	HQ26
MOL005007	Glyasperins M	72.67	0.59	Glycyrrhizae	GC77
MOL004941	(2R)-7-hydroxy-2-(4-hydroxyphenyl chroman-4-one	71.12	0.18	Glycyrrhizae	GC54
MOL004959	1-Methoxyphaseollidin	69.98	0.64	Glycyrrhizae	GC59
MOL000392	Formononetin	69.67	0.21	Glycyrrhizae	GC8
MOL002927	Skullcapflavone II	69.51	0.44	Scutellariae	HQ11
MOL005308	Aposiopolamine	66.65	0.22	Ginseng radix	RS2
MOL004863	3-(3,4-dihydroxyphenyl)-5,7-dihydroxy-8-(3-methylbut-2-enyl) chromone	66.37	0.41	Glycyrrhizae	GC32

**Table 2:** Twenty-two key targets obtained by network topology analysis.

Serial number	Node	Degree	Closeness centrality	Betweenness centrality
1	TNF	83	0.895238095	0.034838308
2	IL6	82	0.878504673	0.026765301
3	AKT1	82	0.886792453	0.029638038
4	IL1B	77	0.839285714	0.017125073
5	TP53	77	0.846846847	0.018483002
6	VEGFA	76	0.831858407	0.013594079
7	MMP9	71	0.796610169	0.019044465
8	JUN	71	0.803418803	0.009314145
9	PTGS2	71	0.803418803	0.013347386
10	PPARG	71	0.796610169	0.022016769
11	CASP3	71	0.796610169	0.009191322
12	CXCL8	69	0.783333333	0.011368298
13	STAT3	68	0.783333333	0.006717204
14	EGFR	68	0.783333333	0.019193115
15	FN1	67	0.770491803	0.011527801
16	EGF	67	0.770491803	0.008309464
17	CCL2	66	0.764227642	0.007120974
18	MYC	65	0.764227642	0.009523735
19	HIF1A	65	0.758064516	0.005326662
20	IL10	65	0.758064516	0.007850675
21	CTNNB1	64	0.752	0.011064031
22	ESR1	63	0.752	0.01743101

**Table 3:** Topological parameters of main active ingredients of XCHD.

MOLID	The active ingredient	Degree
MOL000098	Quercetin	217
MOL000422	Kaempferol	95
MOL000358	Beta-sitosterol	42
MOL002714	Baicalein	36
MOL000449	Stigmasterol	30
MOL000173	Wogonin	28
MOL004328	Naringenin	23
MOL000354	Isorhamnetin	20
MOL002773	Beta-carotene	16
MOL001689	Acacetin	15

**Table 4:** Topological parameters of main targets of XCHD.

The target	Degree
PTGS2	154
HSP90AA1	114
PTGS1	101
AR	97
ESR1	91
NOS2	87
PPARG	82
PRSS1	81
ADRB2	66
DPP4	59

**Table 5:** Molecular docking of XCHD main compounds and HF key targets.

The key target	Major compound	The binding energy/(kcal·Mol^−1^)
PTGS2	Quercetin	− 8.5
	Kaempferol	− 8.3
	Beta-sitosterol	− 3.8

HSP90AA1	Quercetin	− 7.6
	Kaempferol	− 7.5
	Beta-sitosterol	− 7.6

PTGS1	Quercetin	− 7.0
	Kaempferol	− 7.7
	Beta-sitosterol	− 0.9

## Data Availability

The gene expression profifiling data supporting this study are from previously reported studies and datasets, which have been cited.
